# The Use of Helmholtz Resonance for Measuring the Volume of Liquids and Solids

**DOI:** 10.3390/s101210663

**Published:** 2010-11-30

**Authors:** Emile S. Webster, Clive E. Davies

**Affiliations:** 1 School of Engineering and Advanced Technology, Massey University Palmerston North/Riddet Institute (PN 445) Massey University, Private Bag 11 222, Palmerston North 4442, New Zealand; 2 School of Engineering and Advanced Technology, Massey University Palmerston North, P O Box 11 222 Palmerston North 4442, New Zealand; E-Mail: C.Davies@massey.ac.nz

**Keywords:** Helmholtz resonator, volume measurement, density measurement, acoustic measurement

## Abstract

An experimental investigation was undertaken to ascertain the potential of using Helmholtz resonance for volume determination and the factors that may influence accuracy. The uses for a rapid non-interference volume measurement system range from agricultural produce and mineral sampling through to liquid fill measurements. By weighing the sample the density can also measured indirectly.

## 1. Introduction

Helmholtz resonance is the term for resonance occurring in a cavity linked to the surrounding atmosphere via a constricted neck or necks, and is named after Hermann L.F. von Helmholtz [1], who pioneered the discovery of its physical and mathematical principles. Bottles and stringed instruments are perhaps the most familiar examples of Helmholtz resonators, bottles in particular having the characteristic small opening into a large chamber. If air is blown across the neck of a bottle, the air in it resonates at a frequency proportional to the bottle’s dimensions. The fundamental equation for the frequency of an ideal Helmholtz resonator is similar to that for other oscillatory systems such as the classical mass-spring arrangement [2], and is given in most standard textbooks on acoustical theory, see for example Blackstock [3]. Resonant frequency, *f_res_*, is proportional to the speed of sound, *c*, and the square root of the cross sectional area, *s_p_*, of the neck or port, divided by the product of the resonator chamber volume, *V_c_*, and port length, *l_p_*:

(1) $$f_{\text{res}}=\frac{c}{2\pi }\sqrt{\frac{s_{p}}{V_{c}l_{p}}}$$

**Figure 1.**
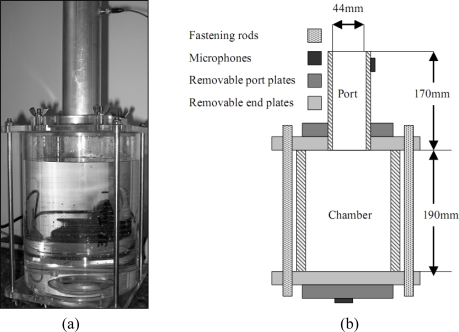
**(a)** Photo of Helmholtz resonator with water fill; **(b)** Schematic of resonator components.

In a real resonator, however, the air within the port oscillates over a distance greater than the physical port length. This was first observed by Lord Rayleigh [4], who proposed an effective port length, *l_p_*’, in which a correction was added to the physical port length to account for end effects. Further refinements have been made by a number of authors which also account for different resonator geometries; see Ingard [5], Alster [2] and Chanaud [6]. Equation (2) is a theoretical expression for the case of a single port located on the axis of the resonator [3]; in acoustics this is sometimes termed an asymmetric port. The term *0.6r* is the correction for the un-flanged end of the port that opens to atmosphere, and the term $\frac{8}{3\pi }r$ is the correction for the flanged end of the port that opens into the interior of the resonator, as indicated in Figure 1:

(2) $$l_{p}^{\prime }=l_{p}+0.6r+\frac{8}{3\pi }r$$

When an object with volume, *V_A_*, is placed in a resonator, *V_A_* reduces the volume of its cavity. Equation (1) can be applied to this situation by replacing *V_c_* vith *(V_c_* *− V_P_)*, in which *V_P_* is the predicted sample volume. After rearrangement, *V_P_* is expressed in terms of the resonant frequency of the system as indicated in Equation (3). Thus, in principle, a measured frequency can be used to determine the volume of an object placed in a resonator of known physical dimensions.

(3) $$V_{P}=V_{c}-\frac{s_{p}}{l_{p}^{\prime }{\left(\frac{{2\pi f}_{\text{res}}}{c}\right)}^{2}}$$

While expressions such as Equation (2) provide significantly improved estimates of *f_res_*, the match between measured and predicted resonant frequencies is not perfect and empirical correction for effective port length is advisable where feasible, see for example, Ingard [5].

Despite extensive historical and ongoing investigations into the physics and understanding of Helmholtz resonators by experimental and theoretical [5–7,2] and numerical approaches [7,8], little has been published on attempts to use a Helmholtz resonator as a measurement device. A preliminary investigation by Nishizu *et al.* [9] indicated successful volume measurements on solids were possible using a Helmholtz resonator in which part of the chamber volume was displaced by a sample volume to be measured; the use of a closed Helmholtz resonator for measuring the volume of a liquid in micro-gravity has been reported by Nakano *et al.* [10].

Potential applications for this technology include: (1) agricultural produce measurements for size and density sorting; (2) dynamic liquid fill level measurements; and (3) mineral extraction and mining in which mineral density and sorting is required. The limitations imposed by the measurement time, when measuring produce samples, may be reduced if the approximate density is known in advance and an estimated volume inferred. Thus, a very localised narrow frequency scan could be performed reducing the measurement time.

In light of the limited prior work into volume measurements using a Helmholtz resonator, the focus in the methods developed in this investigation has been empirical rather than theoretical.

## 2. Experimental Section

### 2.1. Experimental Equipment

An asymmetric single port resonator system was manufactured and could be configured to have nominal internal volumes of 1 L, 2 L or 3 L and a 170 mm long port (Figure 1(a)). An asymmetric port is one in which the port extends outwards from the resonator cavity. All parts except the port were made of clear Perspex™ (150 mm diameter, 5 mm thick walled tube and 12.5 mm thick flats for end plates) to allow easy machining and visibility of samples within the chamber. The port was made of extruded 50 mm aluminium tube with a 3 mm wall thickness giving an internal port diameter of 44 mm. The chamber lengths were 63 mm, 127 mm and 190 mm. Chamber end plates were O-ringed to seal against the chamber tube as well as the port plates. Port plates and chamber end plates were fastened using a combination of threaded rod and threaded studs secured with wing nuts. The advantage of this system was rapid and easy switching of chamber sizes and port configurations.

Two PCB103A sound pressure microphones (PCB Piezotronics Inc. New York, NY, USA) were used to measure resonant frequency and amplitude. The first was spaced 20 mm from the port opening, and the second was centre-mounted in the chamber base (Figure 1(b)). The chamber microphone plate was removed for liquid volume measurements and a replaced with a blank. The PCB microphone amplitude was calculated using the measured voltage signals referenced to a 1 V source to give outputs in decibels (dB). The primary sound source for this investigation was a full range, eight-inch, polycarbonate cone driver in an infinite baffle enclosure. The enclosure was optimally designed using Thiele and Small [11–13] design parameters.

A National Instruments PCI 6221 M series Data acquisition card was used for the signal generation for the acoustic inputs and analysis of the signals from the microphones and temperature sensor. Both generation and acquisition were implemented at 40,000 samples per second, *i.e.*, 40 kHz. Using a high sample rate facilitated smoothly generated fractional sine waves. A 100 W Digitech AA-0470 audio amplifier was used to drive the loudspeaker at a nominal 80 to 90 dB. A resistive temperature device (*RTD*) was used to provide speed-of-sound temperature compensation. Software was designed using National Instruments LabVIEW™ and used to generate and acquire frequency data.

A three-stage hunting algorithm was developed to find and reduce the time required to identify the resonant frequency. First, pink noise was applied to the resonator to establish an approximate resonant frequency, Chanaud [6]. Once determined, a 2 Hz frequency sweep was used to further isolate the resonant peak. Lastly, a very narrow 0.1 Hz sweep was applied to detect the resonant frequency to a precision of 0.005 Hz. The hunting technique reduced the resonant frequency identification time from many minutes, for traditional frequency scanning, to approximately 40 seconds. Quicker times were possible at the expense of accuracy, for example a 20 second hunting time quartered the maximum achievable accuracy.

Regular solid samples, spheres (1 mL to 278 mL) and cubes (2 mL to 864 mL), were used as test specimens. Cubes were precision milled from mild steel; whereas some spheres were made of glass and some of steel. Tap water was used as an exemplar for liquid fill measurements as it was readily available and has well defined characteristics. During volume measurements on water the temperature range was 10 °C to 15 °C. The water was allowed to equilibrate in temperature with its surroundings and de-gas for two hours prior to use. Quantities of water were measured by weight using a set of Mettler PE6000 scales (±0.1 g).

### 2.2. Experimental Method

**Figure 2.**
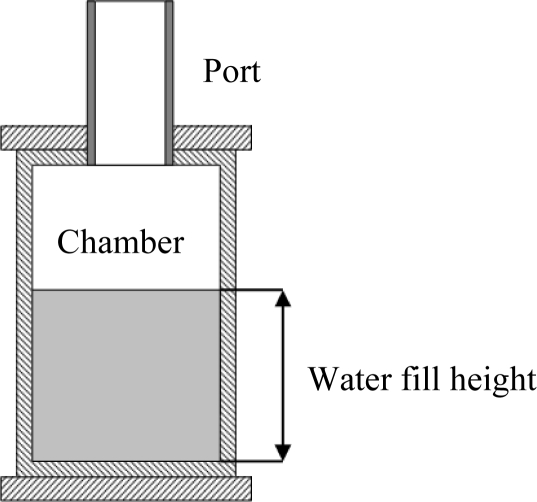
Water filling of resonant chamber.

One hundred mL amounts of water were added to the chamber and the resonant frequencies, Q factors and temperatures recorded (Figure 2). Linear temperature compensation, *c = 331.6 + 0.6θ* [14], was implemented to dynamically adjust the speed of sound constant within the Helmholtz equation, where *θ* is in degrees Celsius. Adjustments for humidity were not made, as its contribution is small and not able to provide any measurement benefits or increased accuracy.

A higher Q factor (Quality factor) for the resonator enables the resonant frequency to be identified more readily and the potential accuracy improved, Equation (4). The Q factor provides an indication of how well the system is resonating. The frequencies *f_1_* and *f_2_* are the roll-off frequencies either side of the main resonant peak and define the narrowness of the peak. By scanning through the frequencies below and above the resonant frequency the *f_1_* and *f_2_* frequencies can be identified:

(4) $$Q=\frac{f_{\text{res}}}{f_{1}-f_{2}}$$

where *Q* is the quality factor, *f_res_* is resonant frequency, *f_1_* is the lower −3 dB frequency, *f_2_* is the upper −3 dB frequency.

A fill level *versus* detected resonant frequency curve could then be plotted and compared to theory using Equation (1). Successful measurement results would allow theoretical back calculation, Equation (3), of a sample’s volume, when placed in the resonator chamber.

Water fill tests were followed by solid sample tests using spheres and cubes to establish how changes in displacement type affect the resonant frequency. All solid samples were centrally located with reference to the port axis on the bottom plate to prevent possible nonsymmetrical acoustic effects within the chamber. Additional work has been undertaken to establish the significance of location, but is not presented in this paper. Again, temperature and frequency were measured and volumes calculated via the modified Helmholtz equation, Equation (3).

## 3 Results and Discussion

### 3.1. Volume Measurements of Water Fill

**Figure 3.**
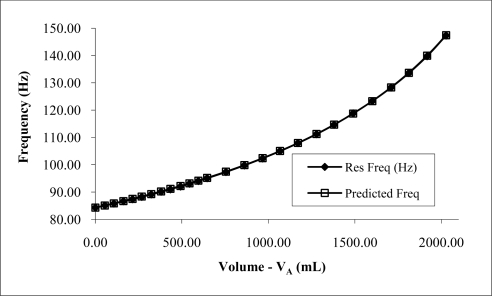
Measured resonant frequency and predicted resonant frequency for varying water fill using 3 L chamber, 170 mm long, and 44 mm diameter port.

**Figure 4.**
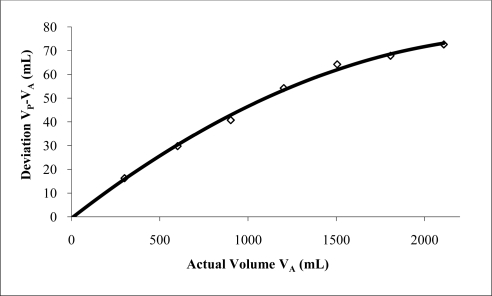
Actual water volume *versus* deviation volume (*V_P_* *− V_A_*) using Helmholtz equation with 3 L chamber, 170 mm long, and 44 mm diameter port.

**Figure 5.**
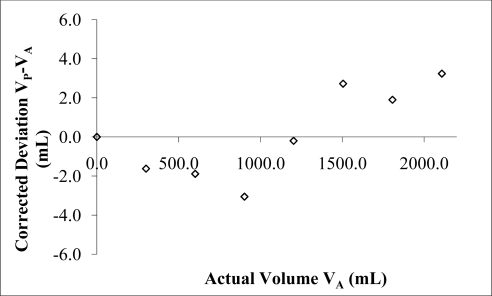
Actual water volume *versus* corrected deviation volume (*V_P_* *− V_A_*) using Helmholtz equation with second order correction. Measured using 3 L chamber, 170 mm long, and 44 mm diameter port.

Initial results using water at different fill ratios revealed measured frequencies close to those predicted using the Helmholtz equation (Figure 3). Results suggested a second order polynomial calibration curve could be applied when comparing the actual water volume (*V_A_*) with the predicted water volume (*V_P_*) using Equation (3) and the deviation volume *V_P_* − *V_A_*. Doing so resulted in a coefficient of determination of 0.99 (Figure 4). Using a parabolic curve fit overlaid on the experimental volume results allowed subsequent measurements to be made within 3 mL of the actual values when measuring water (Figure 5). This represents an accuracy of better than ±0.1% of the resonator’s volume (3 L). Repeatability for a given measurement was generally ±1 mL when successive measurements were made on the same volume.

**Figure 6.**
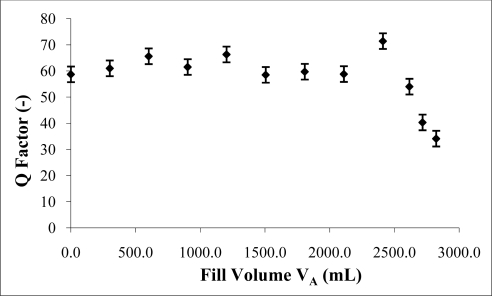
Q factor with increasing water fill level, measured using 3 L chamber, 170 mm long, and 44 mm diameter port.

The reason for a second order deviation is unclear, but could be due to secondary effects caused by implicit assumptions made in simplifying acoustical theory used to generate the Helmholtz equation, see for example Blackstock [3]. These assumptions include the small signal approximations made to allow linearisation and the completion of the wave equation. Also, various small angle approximations are made in lumped parameter analysis within transmission theory. Because frequency measurements were being made to such a high accuracy these seemingly unimportant small terms may now be significant. The Q factor is an important indicator of resonant strength. It was observed that the Q factor remained steady at approximately 60 (Figure 6) up to a fill of 2.5 L in a 3 L chamber.

The success in the predictive capabilities is in part due to the consistently high Q factor. This maintained the resolvability of the resonant peak. Also, the high Q factor is indicative of low energy absorption by the water. At greater fill levels the water approached the interior port where the moving mass of air in the port interferes with the surface of the water. This caused the Q factor to decrease rapidly indicating attenuation in resonance activity, Figure 6. Error bars indicate the range in three repeat measurements of the Q factor value at each successive fill level.

The air in the port moves a physical distance beyond the port entrance and exit determined by the amount of flanging material at each port termination. The extension beyond the port’s physical length is in part also proportional to the inertia of the air contained in the port as it oscillates, [5–7]. The relationship between these variables is not yet completely understood. However, an empirical value can be derived from measurements using different flange sizes and interference caused by a close proximity barrier.

### 3.2. Volume Measurements on Spheres and Cubes

**Figure 7.**
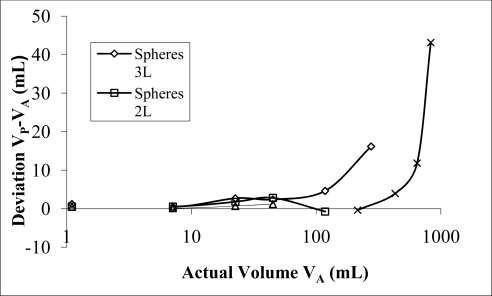
Deviation volume from actual volume for spheres when measured using 1 L, 2 L and 3 L chamber, 170 mm long, and 44 mm diameter port.

Spheres and cubes displayed differences in their effect on the resonant frequency, independently of their equivalent volume displacement. For small displacements both initially have a near flat volume deviation, which rises sharply at a particular displacement (Figure 7). Volume measurements on spheres showed almost one-to-one volume prediction up to ∼100 mL at which point a marked increase in over prediction occurred. The same behaviour was observed in cube samples with over prediction appearing at volumes over 400 mL (Figure 7).

A comparison was also made of data from 1 L, 2 L and 3 L chambers using various spherical samples (Figure 7). There was a visible difference in the volume deviation data between the three chamber sizes used. As noted earlier the 3 L chamber showed a rapid increase in over prediction of the sample volume for the largest samples tested. In contrast the 2 L chamber showed good agreement between predicted volumes and the actual volumes. A local maximum in over prediction is evident, but this may not be significant as the uncertainty in measurement for the 2 L configuration is ±2 mL. The 1 L chamber predicted volume data was also very close to the actual volumes of the samples measured, within 1 mL. Restriction due to the chamber height limited the largest spherical sample that could be tested to 45 mL for the 1 L configuration.

Volume prediction using Equation (3) for cubes and spheres is distinctly different from that observed using water fill. Water tests revealed a volume deviation from prediction, best fitted with a second order polynomial (Figure 4), whereas individual solid samples show a rapid rise in over prediction at a threshold determined by their volume and apparent surface geometry. Over prediction deviations for spheres and cubes were best corrected with an exponential fit. This data suggests that the volume displacement type significantly affects the resonant frequency. The three distinct sample types change the way sound propagates in the chamber and hence the transmission properties of the chamber.

As a pressure wave emanates from the internal end of the port, it encounters a flat surface, in the case of water filling, which is effectively a high impedance barrier. This causes the bulk of the pressure wave to be reflected back up the chamber. If the emanating pressure wave encounters a regular or irregular solid the pressure wave becomes dispersed and the resulting resonant frequency will in part be a function of chamber size and interference. The lumped parameter analysis of the chamber used in deriving the Helmholtz equation will no longer be valid, necessitating a correction curve for accurate volume calculations.

Standard acoustical theory [3], for a sphere, suggests the sound wave is likely to be reflected omni-directionally. In contrast, the cubic samples are liable to reflect the sound waves as point sources from the edges and corners as well as from its planar surfaces. The angular samples represent a different interference source from spherical ones. Adding to the complexity of the angular sample is the size of any flat surfaces. The larger they are the more efficiently they reflect the incident sound pressure waves. Studies conducted by Barmatz *et al.* [15], Leung *et al.* [16] and Cordero and Mujica [17] used rigid spheres in a ½ wave resonant cavity and found scattering to affect the resonant frequency. However, the Helmholtz resonator frequency is related to the chamber volume not a standing wave within the chamber. Therefore, the systems are not directly comparable.

## 4. Conclusions

Volume measurements on specific liquid and solid samples were made to a high accuracy using a suitably designed Helmholtz resonator in which an object to be measured changes the resonator chamber volume. The consequence of this chamber volume change is a measurable change in the induced resonant frequency. Therefore, indirect volume measurements were possible through frequency measurement.

It was established that the volume measurement accuracy was, in part a function of the resonator chamber volume for tested chamber volumes between 1 L and 3 L. A resonant hunting technique, incorporating pink noise and narrow frequency scanning, enabled a reduction in measurement time from several minutes to approximately 40 seconds while maintaining an accuracy of ±0.1% of the chamber volume.

Appropriate calibration curve fitting was required when either solid samples or water-filling displacement was used within the resonant chamber. Water volume displacements could be accurately measured provided a second order polynomial correction was applied and likewise for regular solids using an exponential correction. The Helmholtz resonance equation provides a method for accurately measuring liquids and solids placed within a suitably designed chamber with temperature compensation and a sample-dependant correction curve.

## Nomenclature

*c*: Speed of sound m/s
*f_1_*: Lower −3dB frequency Hz
*f_2_*: Upper −3dB frequency Hz
*f_res_*: Resonant frequency Hz
*l_p_*: Length of port m
*l_p_′*: Port length correction m
*Q*: Quality factor −
*r*: Port radius m
*S_p_*: Port area m^2^
*θ*: Temperature °C
*V_A_*: Actual sample volume mL
*V_c_*: Chamber volume mL
*V_P_*: Predicted sample volume mL

## References

[1] Helmholtz H, Alexander JE (2005). On the Sensations of Tone as a Physiological Basis for the Theory of Music.

[2] Alster M (1972). Improved calculations of resonant frequencies of Helmholtz resonators. J Sound Vib.

[3] Blackstock DT (2000). Fundamentals of Physical Acoustics.

[4] Rayleigh JWS (1945). The Theory of Sound, Volume Two.

[5] Ingard U (1953). On the theory and design of acoustic resonators. Acoust Soc Amer.

[6] Chanaud RC (1993). Effect of geometry on the resonant frequency of Helmholtz resonators. J Sound Vib.

[7] Selamet A, Lee I (2003). Helmholtz resonators with extended neck. Acoust Soc Amer.

[8] Kang ZX, Ji ZL (2007). Acoustic length correction of duct extension into a cylindrical chamber. J Sound Vib.

[9] Nishizu T, Ikeda Y, Torikata Y, Manmoto Y, Umehara S, Mizukami T (2001). Automatic, continuous food volume measurement with a Helmholtz resonator. CIGR J Sci Res Dev.

[10] Nakano A, Torikata Y, Yamashita T, Sakamoto T, Futya Y, Tateno A, Nishizu T (2006). Liquid volume measurement with a closed Helmholtz resonator under micro-gravity conditions. Cryogenics.

[11] Thiele AN (1971). Loudspeakers in vented boxes, Parts I. J Audio Eng Soc.

[12] Small RH (1972). Direct-radiator loudspeaker system analysis. J Audio Eng Soc.

[13] Small RH (1972). Closed-box loudspeaker systems, Part I. J Audio Eng Soc.

[14] Kinsler LE, Frey AR (1962). Fundamentals of Acoustics.

[15] Barmatz M, Allen JL, Gaspar M (1983). Experimental investigation of the scattering effects of a sphere in a cylindrical resonant chamber. Acoust Soc Amer.

[16] Leung E, Lee CP, Jacobi N, Wang TG (1982). Resonant frequency shift of an acoustic chamber containing a rigid sphere. Acoust Soc Amer.

[17] Cordero ML, Mujica N (2007). Resonant frequency shifts induced by a large spherical object in an air-filled acoustic cavity. Acoust Soc Amer.

